# Can Multidrug‐Resistant and Extensively Drug‐Resistant Tuberculosis be Treated More Successfully?

**DOI:** 10.1002/puh2.70363

**Published:** 2026-09-15

**Authors:** K. L. Holloway‐Kew, M. Henneberg

**Affiliations:** ^1^ Deakin Institute for Mental and Physical Health and Clinical Translation (Deakin IMPACT) School of Medicine Deakin University and Barwon Health Geelong Victoria Australia; ^2^ School of Medicine Adelaide University Adelaide South Australia Australia; ^3^ Institute of Evolutionary Medicine University of Zurich Zürich Switzerland

**Keywords:** extensively drug‐resistant, multidrug‐resistant, tuberculosis

## Abstract

**Background:**

Signs and symptoms of tuberculosis (TB) are a result of a disturbed balance between *Mycobacterium tuberculosis* and the patient's tolerance to its pathogenic properties. Pharmacological treatments elicit the evolution of drug resistance, and there is a need to consider alternative treatment methods. We performed a statistical analysis of World Health Organization (WHO) data, combined with an analysis of old medical records, aimed at suggesting a new treatment.

**Methods:**

Data from the WHO for all countries worldwide (*N* = 215) on the epidemiology of all TB cases, human immunodeficiency virus (HIV)‐related TB, multidrug‐resistant TB (MDR) and extensively drug‐resistant TB (XDR) in 2012–2022 contain prevalence, success rate of treatment, mortality and numbers of failed treatments and patients lost‐to‐follow‐up. Data on success rates in old ‘sanatoria treatment’, widespread before the 1940s, were used to predict hypothetical success rates of drug‐resistant cases if sanatoria treatment were added to treatment regimes.

**Results:**

After 2012, the number of MDR and XDR cases increased faster than the total number of TB cases, as did their proportions among the total. Success rates of MDR (∼70%) and XDR (around 60%) treatments would hypothetically increase to around 90%, when supplemented by sanatoria treatment success.

**Conclusions:**

Adding treatments for increasing tolerance to TB infection to the current pharmacological treatments may improve outcomes.

## Graphical abstract

Drug‐resistant cases of tuberculosis are increasing, complicating treatment strategies.

Prior to the development of pharmacological agents, improving patient health at institutions called ‘sanatoria’ was a successful primary treatment.

World Health Organization data combined with information from historical reports estimate that current treatments can be improved by adding ‘sanatoria‐like’ treatment.

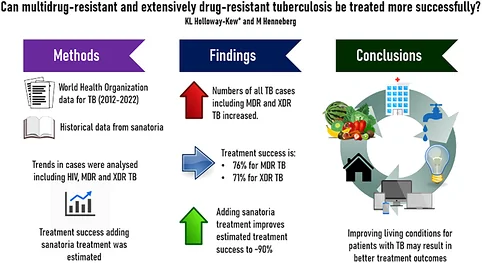

## 1 Introduction

Tuberculosis (TB) is a widespread infectious disease that has been in human populations for at least 10,000 years [1, 2]. During this time, a coevolution of the pathogen and human host occurred, leading to a lower virulence of the disease; only approximately 10% of individuals infected with *Mycobacterium tuberculosis* experience pathological signs and symptoms [1, 3]. Many humans are now considered patients with ‘latent TB’, and when tested for the presence of the pathogen, they return positive results, but they feel well. Had they not been tested, they would have no way of knowing they are ‘ill’. These people may develop signs of TB if their immunity becomes compromised by poor living conditions or a separate disease.

Robert Koch, when discovering the cause of TB, clearly stated in his ‘postulates’ that infection with *M. tuberculosis*, or similar, causes the disease. Bradford Hill epidemiological criteria statistically link a cause with a disease, measuring an association between the presence of a cause and the appearance of a disease. Association, however, does not prove causality in the sense that every time the cause occurs, the disease must appear. It is increasingly considered that a disease results from an altered dynamic balance between the pathogen and the body's ability to control the results of the infection. In this understanding, to cure a disease, it may not be necessary to remove the pathogen but to strengthen the body's control over the pathological effects of infection [4]. An example of how the overall body's immunity influences the manifestation of a disease is the greater incidence of TB in patients with human immunodeficiency virus (HIV). Their immunity is suppressed, and TB signs and symptoms manifest among them in greater numbers than among the general population in which they live [5].

In the past, when pharmacological remedies for a disease were not known, strengthening the body's mechanisms to control disease was practised [6]. The simplest approach was to adopt a ‘bed rest’ regimen, limiting exhausting physical activities, ensuring good nutrition and maintaining body warmth. In the early years of industrialisation, the prevalence of TB increased because industrial labourers lived in crowded urban conditions with inadequate food supply and worked very long hours [7]. Developed countries began implementing public health policies to reduce TB incidence in the 19th century, which significantly reduced TB mortality [7, 8, 9]. These policies comprised better accommodation, nutrition and lifestyle, providing improved tolerance and resistance to TB. There were also measures limiting the contact of patients manifesting TB signs with others and special facilities where patients were provided optimal living conditions and high‐protein nutrition. These latter facilities were known as ‘sanatoria’.

TB mortality declined 10‐fold in many European and North American countries from about 1800 to 1945 before effective pharmacological TB treatments became available (approx. 600/100,000–60/100,000 [9, 10]). Such improvement was possible due to the general economic development and the application of specific rules concerning the treatment of TB patients. The success of the sanatorium treatment has been described by Rucker and Kearny [11], reporting that 62.6%–96.7% of patients improved, depending on the severity of the disease upon entry into the sanatorium. Many of these patients were able to return to work after leaving the sanatorium (60.4%–97.0%).

The introduction of effective pharmacological treatments after 1945 further reduced TB and became a standard approach to treatment. Antibiotics that become widespread pharmaceuticals, although largely effective, have a weakness. As antibiotics are substances originally produced by living organisms, they are not potent toxins affecting the basic metabolism of microorganisms, but interfere with pathogen properties less crucial for their survival. These properties are modifiable by mutations. As expected in any biological system, antibiotics, through altering mutation/selection balance, result in the evolution of pathogenic mycobacteria strains resistant to standard pharmacological treatments—isoniazid and rifampicin [12]. These antibiotic‐resistant strains are now resulting in a resurgence of TB. The evolution went so far that there are now cases of multidrug‐resistant (MDR) and extensively drug‐resistant (XDR) TB.

Due to the assumption that the only cause of TB is *M. tuberculosis*, modern treatments for antibiotic‐resistant TB consist of new antibiotics aimed at the removal of the pathogen. These are fairly expensive to produce and apply, whereas their effectiveness is lower than ‘old’ antibiotics. Like with any pharmacological treatment of TB, the treatment of MDR and XDR patients is prolonged, making it difficult to encourage their adherence. Placement of drug‐resistant patients in hospitals should address nonadherence and expose them to sanatorium‐like conditions. Hospital patients are typically resting in beds, receiving optimal nutrition designed by dietitians and undergoing ongoing general healthcare by clinicians. It seems, however, that hospitalisation of all drug‐resistant TB patients for months is challenging to introduce because of the significant cost of hospitalisations and the limited number of hospital beds, especially in less affluent populations. Hospitals have to be equipped and staffed to deal with an extensive range of health problems, which increases the cost of TB patient's hospitalisations beyond the economic abilities of individuals and health service systems.

We are in a situation of a ‘race’ between evolving resistant strains of *Mycobacterium* and the ingenuity of people inventing and producing new drugs for new treatment regimes. From an evolutionary perspective, the outcome is inevitable—newly evolving strains of *Mycobacterium* will prevail. This, however, does not necessarily mean that we will not be able to limit the impact of TB on human health and mortality by improving our bodies’ tolerance to *Mycobacterium* infection.

The aim of this article is to investigate the outcomes of current MDR and XDR treatments aimed at removing *Mycobacterium* from patients’ bodies and consider how the introduction of public health measures, including sanatoria treatment, may improve these outcomes.

## 2 Methods

Data on TB case numbers, as well as treatment outcomes for the years 2012–2022 for 215 countries, were obtained from the World Health Organization (WHO) [13]. Data files downloaded from the WHO included ‘*Case notifications’* and ‘*Treatment outcomes’*. Data for HIV, MDR and XDR TB were only available until 2020.

### 2.1 Statistical Analyses

Distributions of data per country were analysed by calculating their measures of central tendency and standard deviations. Kruskal–Wallis tests were used to examine changes across the year range studied (2012–2022) for: case numbers, treatment success and failure rates, mortality and loss to follow‐up. These analyses were completed separately for (i) all TB cases, (ii) TB cases with HIV, (iii) MDR TB and (iv) XDR TB.

In an exploratory analysis, success rates of treatments for MDR and XDR TB and their mortality rates were further altered by correcting them for additional success were sanatoria treatment used in addition to standard treatments. This was done by combining data from multiple reports [11, 14, 15, 16, 17, 18] describing the proportion of patients that ‘improved’ following sanatorium treatment (65.5%, Supplementary S1).

$$\mathrm{New}\;\mathrm{success}\;\left(\%\right)=\%\mathrm{success}+\left(100\%-\%\mathrm{success}\right)\times 0.655$$

$$\mathrm{New}\;\mathrm{mortality}\;\left(\%\right)=\mathrm{mortality}\%\times \left(1-0.655\right)$$

Analyses were completed using Stata (Version 17. Stata Corp. 2017. Stata Statistical Software: Release 17. College Station, TX: StataCorp LLC).

## 3 Results

### 3.1 TB Case Numbers

**FIGURE 1:**
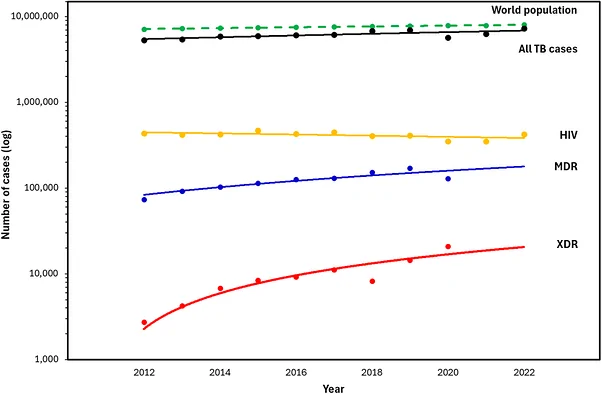
World numbers of tuberculosis (TB) cases, including all cases (black), cases with human immunodeficiency virus (HIV, yellow), multidrug‐resistant (MDR, blue) and extensively drug‐resistant (XDR, red) cases, across the years 2012–2022. The world population size, in thousands, is also presented (green). Note that no data were available for MDR/XDR cases in 2021 or 2022.

Worldwide, the number of all TB cases (*p* = 0.006), as well as MDR (*p* < 0.001) and XDR (*p* < 0.001) cases, increased over the period investigated (Figure 1). However, the number of HIV‐related cases decreased somewhat from 2012 to 2022 (*p* = 0.060).

### 3.2 Proportions of HIV, MDR and XDR Among All TB Cases per Country

**FIGURE 2:**
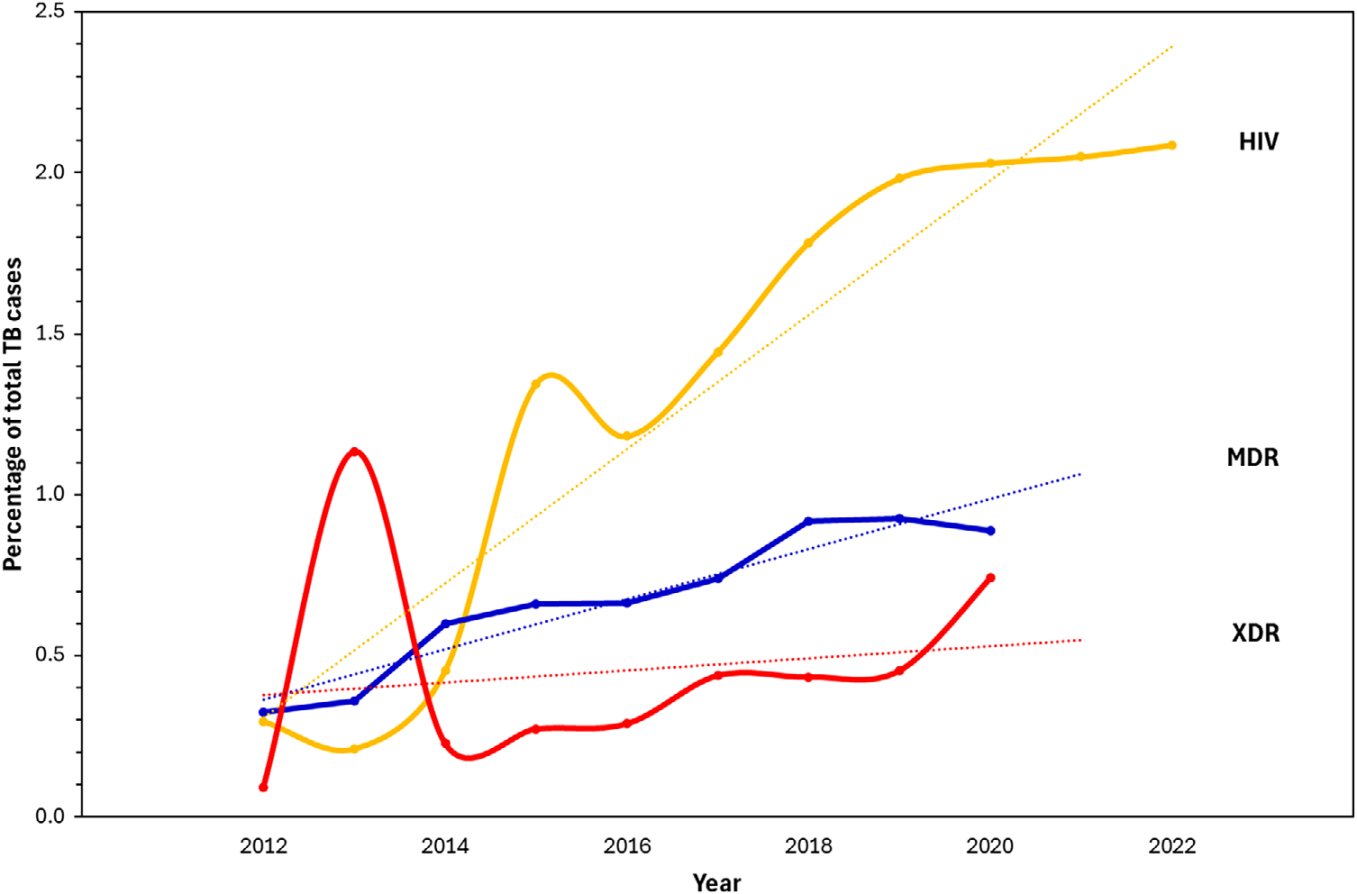
Proportions of human immunodeficiency virus (HIV, yellow), multidrug‐resistant (MDR, blue) and extensively drug‐resistant (XDR, red) TB cases as a percentage of the total number of tuberculosis (TB) cases in the world, across the years 2012–2022. Note that no data were available for MDR/XDR cases in 2021 or 2022. Median values are shown for HIV and MDR cases. Mean values are shown for XDR cases because median values were zero for all years due to low numbers. Sample sizes for each year of HIV oscillate between 109 and 139 countries, for MDR between 131 and 156 countries and for XDR between 45 and 80 countries. Other countries did not provide information to the World Health Organization.

The average proportions of TB cases with HIV among all TB cases increased across the period studied, from a median of 0.3% in 2012 to 2.0% in 2022 (Figure 2, *p* = 0.039). The proportions of MDR cases among the total TB cases also increased from 2012 to 2020 (Figure 2, *p* < 0.001). For XDR cases, median values were zero for all years due to few countries reporting XDR cases. Mean values for the proportion of XDR cases out of all TB cases rose from 0.09% to 1.10%.

### 3.3 Treatment Outcomes

**FIGURE 3:**
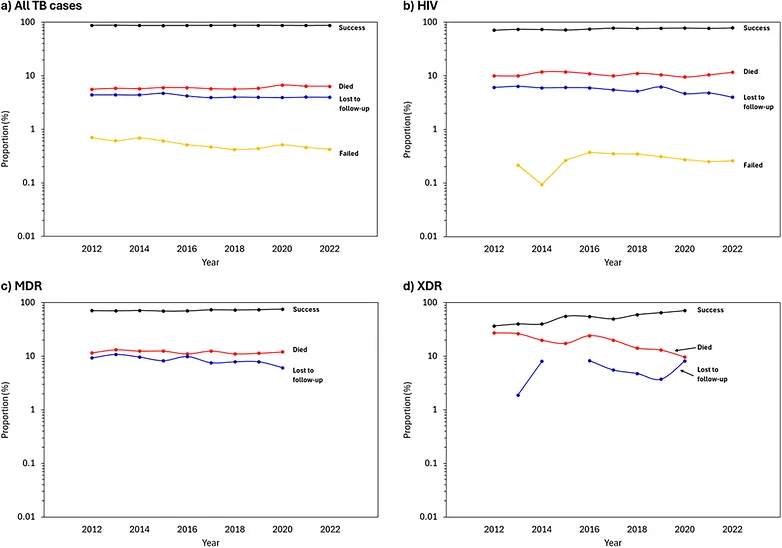
Median treatment outcomes (success, failure, death and lost‐to‐follow‐up) for all countries of the world that reported information to the World Health Organization (WHO) for (a) all tuberculosis (TB) cases (187–198 countries each year), (b) cases with human immunodeficiency virus (HIV) (109–139 countries each year), (c) multidrug‐resistant (MDR) (131–156 countries each year) and (d) extensively drug‐resistant (XDR) (45–80 countries each year) cases over the years 2012–2022. Data presented as proportion (%) of the total numbers of cases of each category. Note that no data were available for MDR/XDR cases in 2021 or 2022. Median values for ‘failed’ treatment were zero in 2012 for TB cases with HIV, and for MDR and XDR cases across all years. ‘Lost to follow‐up’ median value was also zero for XDR cases in 2012 and 2015. Note that the ordinate (y) scale is logarithmic.

The average treatment outcomes for all TB cases, HIV, MDR and XDR are shown in Figure 3. For all TB cases, there were no changes in success, death or loss to follow‐up from 2012 to 2022 (all *p* > 0.05). However, there was a decrease in the proportion of failed treatment outcomes (*p* = 0.043).

For TB cases with HIV, the success rate increased from a median of 71.4% in 2012 to 79.1% in 2022 (*p* < 0.001).

There were no changes in failed, death or loss to follow‐up outcomes for MDR cases across the period studied (all *p* > 0.05). However, successful treatment outcomes did increase from 71.1% in 2012 to 76.0% in 2020 (*p* < 0.001). For XDR cases, there was an increase in treatment success, from a median of 36.8% in 2012 to 71.4% in 2020 (*p* < 0.001). There was also a decrease in deaths of XDR cases, from a median of 27.3% in 2012 to 9.7% in 2020 (*p* < 0.001).

### 3.4 Estimated Success Rates for Multidrug‐Resistant TB and Extensively Drug‐Resistant TB When Sanatoria Treatment Is Included

**FIGURE 4:**
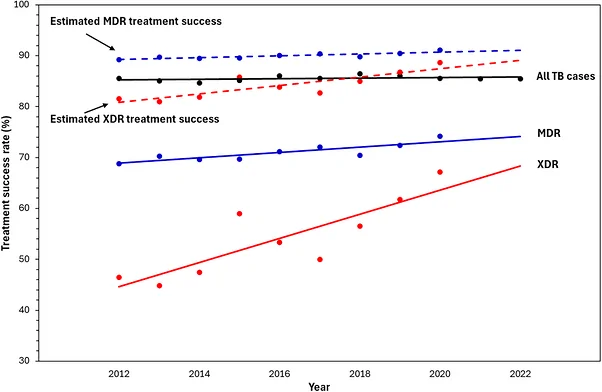
Average estimated success rate with sanatoria treatment for multidrug‐resistant (MDR, blue) and extensively drug‐resistant (XDR, red) tuberculosis (TB), dashed lines. The actual treatment success rates for all TB cases (black) and MDR and XDR cases (blue and red lines) are also shown. Note that no data were available for MDR/XDR cases in 2021 or 2022.

Estimating the percentage of additional successful outcomes through the inclusion of sanatoria treatment (Figure 4) shows that the success rates of MDR and XDR TB treatment would hypothetically rise to become the same as the current success rate of treatment of all TB cases.

Similarly, a very rough estimation of the improvement of the mortality rates of MDR and XDR TB by the lower success rate of sanatoria treatments would bring these rates down to 5.5%, roughly comparable to current all TB death rates. Of course, our estimates would be even better if we took into account success rates of sanatoria treatments improved by developments in nutrition and general health care over the last 100 years.

## 4 Discussion

This study has shown that the current treatment outcomes are still poorer for HIV, MDR and XDR than for all TB cases. Most of the HIV‐related cases are caused by drug‐susceptible *Mycobacteria*, which should be theoretically as responsive to standard treatment as in other TB cases. They, however, occur in people of pathologically lowered immunity, often living in poor circumstances. These factors lower the success rates of HIV‐related cases. The difference between all TB success rates and those of HIV‐related cases indicates the role of lowered immunity in the prevalence of TB. Treating drug‐resistant cases uses the common principle—pharmacological removal of the *Mycobacterium*—as is the treatment of all TB cases. The difference lies in the kinds of specific substances used. These seem to be less effective against drug‐resistant *Mycobacterium* variants. As with all TB pharmacological treatments, the dosage of pharmaceuticals must continue over long periods. This is mainly done in ambulatory situations. The regularity of patients’ visits for treatment is difficult to manage, given the numerous challenges they face in their daily lives. Therefore, considerable nonadherence enables further evolution of drug resistance.

Given that many humans have had or have a commensal relationship with *Mycobacterium* (latent TB patients) in the past and present, it is worth considering how their circumstances could be reintroduced to restore health (=normal organismal homeostasis) in patients where pharmacological treatments have been ineffective. Supportive care strategies, such as public health measures and sanatoria treatment, should be explored to improve the health of communities. Like with many noncommunicable diseases, whose causes cannot be eliminated because they are genetic or systemic, the management of TB should aim at ensuring good patient homeostasis, providing a normal quality of life if complete eradication of its microbial causative factor is not possible.

Sanatoria treatment can assist in this objective in two ways; (i) aiding recovery of those with active disease and (ii) reducing transmission through isolation. Previous studies have reported on the effectiveness of sanatoria treatment; however, not all report outcomes in the same manner. Some provide only ‘cure’ and/or mortality rates, whereas others provide more detailed descriptions of the patients following discharge, such as their ability to perform work. Despite this heterogeneity, these outcomes can be summarised into ‘improved’, ‘not improved’ and ‘died’. Considering multiple reports [11, 14, 15, 16, 17, 18], the averages for these categories are 65.5% improved, 26.3% not improved and 6.5% died. Although these previous reports are heterogeneous in countries of origin, years, and the patient's stage of disease at admission, overall, it can be estimated that approximately two‐thirds of patients showed improvement in their physical condition following sanatorium treatment.

Data regarding readmission and relapse have also been reported as 7.7% [19] and 4.0% [17], respectively. Additionally, mortality data after discharge are available for three different groups of patients followed over several months [14]: 31.9% died after 9 months, 27.8% after 3 years and 44.5% after 6 years. It is important to note that following discharge, these patients would likely have returned to poor living conditions, which may have contributed to an increase in mortality. This is an important consideration following the completion of treatment; sanatoria cannot provide any further assistance once the patient has been discharged [14]. In the past, sanatoria treatment was used to improve living conditions for individuals diagnosed with TB. This improvement in living conditions is what we are highlighting as a method for aiding the treatment of TB, particularly for patients with MDR or XDR. Sanatoria treatment, though efficient to a degree, needs to be combined with strengthened social and supportive care, as well as pharmacological interventions.

Several publications have reported the ability of patients to return to work following discharge from sanatoria treatment [11, 14, 17]. Their results can be summarised into average values for able to work (69.4%), not able to work (22.4%) and died (8.0%). Overall, these reports from the late 1800s and early 1900s indicate that sanatoria treatment was effective, with approximately two‐thirds of patients improved enough to return to work.

Part of the success of sanatorium treatment comes from improving the nutrition of patients, which increases their ability to maintain homeostasis. Underweight individuals are more likely to develop more severe active TB and are also less likely to be treated successfully [20, 21]. Consequently, multiple recommendations for tackling the burden of TB have included addressing undernutrition and low body weight [22, 23]. Simulations including nutritional supplementation for undernourished individuals in India indicated that such a strategy would be cost‐effective in reducing TB incidence and mortality [24]. Thus, it may be expected that sanatorium treatment, including better diet, could enhance treatment outcomes, particularly for undernourished individuals. Over the last century, the knowledge of nutrition and treatment of many co‐morbidities of TB has improved significantly. This indicates that nutrition, living conditions and medical treatments in modern sanatoria would provide greater success of TB treatment than that observed a century ago.

Our exploratory estimates of the effects of sanatoria‐style treatments indicate that success rates of treating patients with drug‐resistant TB would bring them to a level similar to success rates of drug‐susceptible TB. Of course, the ‘success’ in sanatoria treatment will consist of removing TB signs and symptoms, not eradicating *Mycobacteria*. Nevertheless, so‐treated people will be fully capable of conducting normal lives instead of dying or suffering a serious disability.

There is, of course, a cost related to the sanatorium treatment. It is, however, small compared to hospitalisation. Sanatoria treatments in the past were not aimed at treating all kinds of diseases—they were focused on improving the lives of TB patients. Sanatoria were preferably located in situations of low population density, clean air and exposure to sunlight and provided patients with high‐protein diets, rest and light exercise. As sanatoria treatment improved the general resistance and disease tolerance for patients, some of them, even after the introduction of pharmacological TB treatments, were retained in some countries and are used today to improve the well‐being of convalescents from various health problems.

In Poland, sanatoria treatments are now used for giving rest and recovery to patients of all kinds of conditions unrelated to TB. The 2024 prices for accommodation and nutrition at Polish sanatoria are available [25]. Depending on the quality of a particular sanatorium's accommodation (single or multiple patient rooms, bathroom access, etc.) and the season, the cost per day varies between USD4.1 and USD15.9 at 2024 exchange rates. Poland has a high‐income, industrialised, developed economy that ranks fifth in the European Union by GDP, including extensive public services characteristic of developed economies. The quality of Polish health services is adequate in terms of clinical standards, cleanliness and security in the judgement of one of the authors, who, earlier in their life, was successfully treated for TB there. The Polish health system runs current ‘recovery sanatoria’ consisting of a number of dormitories, dining rooms and gardens located in ‘vacation resorts’, small towns or villages with clean air and lots of nature trails to walk.

The actual cost of using TB sanatoria treatments in Britain at the beginning of the 20th century was comparable to Polish figures. The number of GBP Latham [26] quotes per day (0.24) at the 1906–2024 depreciation rate [27] gives 3.66 GBP per day, which converts to USD4.80 today. The cost of sanatorium accommodation and nutrition may be somewhat less in countries with weaker economies. To this cost must be added the cost of medical treatments and personnel, which will include some nurses and medical practitioners, but in much lesser numbers than in hospitals. It is also possible to use home treatment of TB for individuals who do not wish to be treated at a sanatorium [28]. This treatment needs to include regular home visits from medical staff, financial and nutritional support. In resource‐limited settings, these components of treatment need to be efficient, scalable and accessible in order to have a significant impact on treatment outcomes, especially for those with MDR or XDR TB. Home‐ or community‐based treatment of TB may be preferable for patients, particularly with regard to the stigma of hospital treatment and avoiding long periods of isolation. However, even in home‐based treatment, regular visits from healthcare workers can be stigmatising and disempower patients [29], though such visits are necessary to ensure patients’ adherence to treatment, which tends to decrease in the home environment due to immediate pressures of family and social responsibilities.

Several recent studies have investigated the efficacy of patient‐centred care for individuals with drug‐resistant TB. For example, Adepoyibi et al. [30] examined a pilot model of patient education and counselling for individuals with drug‐resistant TB from Papua New Guinea. The study reported that the pilot model led to a high retention in treatment; however, it required a significant amount of resources, highlighting the need for cost‐effective scalability of the model. A recent meta‐analysis [31], including 20 controlled trials, also supports patient‐centred care for TB treatment, reporting that community‐based and digital interventions, along with support for other factors such as nutrition, finance and education, can substantially improve outcomes. Importantly, patients may prefer community‐based patient‐centred care approach, which can improve treatment adherence and subsequent outcomes. One study by Makabayi‐Mugabe et al. [32] showed that patients from Uganda with MDR TB preferred to complete their treatment at home with the support of a community health worker. Patients preferred to take their medication at home for multiple reasons, including to save time and money, while also avoiding some of the stigma surrounding the disease. Additionally, patients found the community health workers knowledgeable and felt that they could also provide psychosocial support where needed. However, the study did note that although the patients preferred this method of treatment, its effectiveness still needs to be evaluated. Another study by Law et al. [33] also reported similar findings, which included conducting interviews with 24 patients (11 with drug‐resistant TB) treated in 11 different countries across the WHO regions of Africa, America, Eastern Mediterranean, Europe, South‐East Asia and the Western Pacific. All patients in the study reported at least one comorbidity and all experienced socioeconomic hardship. The results showed that undertaking TB treatment worsened the symptoms and management of comorbidities and, in some cases, led to further health problems, including poor mental health. As patients may have multiple potentially competing priorities for healthcare, a patient‐centred model, aimed at ensuring appropriate treatment of TB as well as other comorbidities, is required to reduce the morbidity and mortality associated with the disease.

The results of this study align with the current multisectoral accountability framework (MAF) developed by the WHO, with the goal to end TB by 2030 [34]. The MAF describes how a significant reduction in TB incidence and prevalence cannot be achieved through improvements in healthcare alone [35]. The burden of TB is influenced by many factors, including poverty, nutrition, access to clean water, pollution, inequality and inappropriate housing, all of which need to be simultaneously targeted in a combined multisectoral effort to reduce the incidence and prevalence of the disease and improve treatment outcomes [34]. The MAF for TB involves four key principles: Commitments, Actions, Monitoring and Reporting and Review, which are informed by multiple WHO publications, including the Sustainable Development Goals for 2030 and WHO's End TB Strategy [34]. The Framework includes a variety of activities across multiple sectors including development of legislation and health coverage policies, addressing social determinants, strengthening health systems, improving media coverage, funding and research, as well as reporting TB cases and outcomes according to WHO guidelines at an appropriate level of quality and coverage. The Review of these activities requires high‐level leadership and should involve representatives from multiple sectors and other relevant groups such as sectors responsible for finance, poverty, social determinants, housing, labour, justice, migration, science and education, as well as stakeholders such as local governments, private sectors and TB‐affected communities [34, 35]. Several countries have already provided examples of the MAF for TB being implemented, highlighting the challenges they faced, including funding, resources, organising the high‐level leadership required and setting up appropriate systems for monitoring and reporting [36]. Our study supports the objectives of the MAF for TB; we advocate the organised improvement of living conditions with the aid of patient‐centred care, for the better treatment of TB, particularly for patients with drug‐resistant disease. This improvement can also help reduce the cost and burden to the patient, as well as maintain cure or remission after treatment completion. Without these improvements, the situation would be similar to what often occurred in the past with sanatoria treatment, where a patient would improve, then be sent home to poor living conditions which could lead to the disease becoming active again.

In addition to these guidelines for the MAF at a country‐ and global‐level, the WHO has also provided guidelines for the treatment of drug‐resistant TB at a patient‐level [29]. These recommendations are also supported by this study, highlighting the importance of improving a patient's environment to increase the chances of successful treatment. The guidelines indicate that in addition to pharmacological treatment, other supports can be provided, including patient education, social and material support and tracers such as reminder messages or telephone calls. However, it is noted that these supports need to be provided equitably, otherwise inequalities in health will increase. Additionally, although these supports can assist the individual, a patient with TB is usually not living in isolation and may share materials such as food with their community rather than consume it themselves. Therefore, the larger scale MAF for TB approach is needed to effectively reduce the burden of TB and improve treatment outcomes.

This study has some strengths and limitations. The data used in this study are from a reliable source (WHO); however, the quality of data reported or estimated by various countries in this source is varied. It is also unclear what the term ‘success’ or ‘failure’ refers to in WHO files. A further limitation is the relative scarcity of information on the success rates of sanatorium treatments because these were collected a long time ago, assessed by varied criteria and published in difficult‐to‐access sources. However, irrespective of the approximative nature of the data used, it is obvious that increasing resistance and tolerance to TB infection can reduce its mortality and health effects. The application of the evolutionary perspective is a strength.

## 5 Conclusions

We must pay more attention to promoting pathogen resistance and tolerance that will support health by maintaining good organismal homeostasis. Research on this approach has been largely neglected in favour of efforts to develop pharmacological methods for killing pathogenic germs, which unfortunately elicit the germs’ evolution. The ambitious goal of eliminating all germs worldwide is faltering on multiple fronts. It has recently been defeated by rules of biological evolution governing the COVID‐19 pandemic.

## Author Contributions

**K. L. Holloway‐Kew**: conceptualisation, data curation, formal analysis, investigation, visualisation, writing – original draft, writing – review and editing. **M. Henneberg**: conceptualisation, data curation, formal analysis, investigation, methods, visualisation, writing – original draft, writing – review and editing. The corresponding author attests that all listed authors meet authorship criteria, and that no others meeting the criteria have been omitted. All authors have approved the final submitted manuscript.

## Funding

The authors have nothing to report.

## Ethics Statement

This study uses official World Health Organization data, as well as data previously studied and published by several authors. No individual patients’ data, nor particular samples, were analysed. All data used here were anonymised.

## Consent

The authors have nothing to report.

## Conflicts of Interest

The authors declare no conflicts of interest.

## Supporting information




**Supporting File 1**: puh270363‐sup‐0001‐SuppMat.docx.

## Data Availability

This study uses official World Health Organization data, as well as data previously studied and published by several authors. No individual patients’ data, nor particular samples, were analysed. All data used here were anonymised.
