# Supplementary material for: Can Multidrug‐Resistant and Extensively Drug‐Resistant Tuberculosis be Treated More Successfully?
Source: Public Health Chall. 2026 Sep 15;5(3):e70363. doi: 10.1002/puh2.70363 (PMC13577933; doi:10.1002/puh2.70363)
Supplement: Supplementary file 1 [file PUH2-5-e70363-s001.docx]

Supplementary S1: Summary of treatment outcomes for sanatoria treatment.

| **Citation** | **Sanatorium** | **Additional notes** | **Reported treatment outcomes** | **Percentage of patients that improved** |
| --- | --- | --- | --- | --- |
| Ascoli (1912)(1) | Pianosa Island, Italy | This sanatorium treated criminals | 13.0% recoveries | **13.0%** |
| Ascoli (1912)(1) | Hospital on the island of Santa Maria della Grazia (opened 1906), Italy |  | During first two years, 195 of 297 patients under treatment were discharged with improved health | **65.7%** |
| Curran (1942)(2) | Multiple (Ireland) | These reported outcomes were for approximately six months of treatment | Of the 344 patients discharged in 1940, treatment outcomes were: 116 (33.7%) condition unchanged, 32 (9.3%) worse, 12 (3.5%) died, 135 (39.2%) improved, 49 (14.2%) quiescent | **53.5%** |
| Eylers (2014)(3) | Dannenfels, Germany | Patients were treated during 1893-1898, for six months to one year | Treatment outcomes were:   - Healed, [fit] to resume work; 21% - Much improved, almost healed, able to resume work; 16% - Improved, able to work; 12% - Improved, able to work until readmission to Dannenfels; 4% - Left Dannenfels before being cured; 15% - Unimproved; 25% - Died; 4% - Still under treatment; 3% | **53.0%** |
| Lawson (1908)(4) | Multiple (Germany) | Data are from 1899-1903 | Percentage of patients considered cured for each year were:   - 1899, 74% - 1900, 72% - 1901, 77% - 1902, 78% - 1903, 80% | **76.2%** |
| McCrindle (1912)(5) | Multiple (England) |  | Of 126 patients, discharge outcomes were: 80 (63.5%) arrested, 20 (15.9%) much improved, 16 (12.7%) improved, 10 (7.9%) not improved | **92.1%** |
| Porter (1921)(6) | Multiple (Canada) | These sanatoria treated ex-service men | 6,636 of 8,676 patients had been discharged, some cured, others quiescent | **76.5%** |
| Rucker & Kearny (1913)(7) | Swiss sanatoria – Patients admitted in the ***first*** stage of the disease | Data for patients discharged between 1905 and 1911 | 96.7% improved, 3.2% unimproved, 0.1% died | **96.7%** |
| Rucker & Kearny (1913)(7) | Swiss sanatoria – Patients admitted in the ***second*** stage of the disease | Data for patients discharged between 1905 and 1911 | 86.6% improved, 12.4% unimproved, 1.0% died | **86.6%** |
| Rucker & Kearny (1913)(7) | Swiss sanatoria – Patients admitted in the ***third*** stage of the disease | Data for patients discharged between 1905 and 1911 | 62.6% improved, 34.4% unimproved, 3.0% died | **62.6%** |
| Warren (2006)(8) | Falkenstein, Germany |  | 4-4.5% mortality, 14% cures, 45% ameliorations | **59.0%** |
| Warren (2006)(8) | Gorbersdorf, Germany |  | 7.51% mortality, 25% cures, 50-55% ameliorations | **77.5%** |
| Warren (2006)(8) | ” (Rempler), Germany^a^ |  | 7.5% mortality, 25-27% cures, 50% ameliorations | **76.0%** |
| Warren (2006)(8) | ” (Ruchler), Germany^a^ |  | 4.0% mortality, 72% ameliorations, 24% not ameliorated | **72.0%** |
| Warren (2006)(8) | Reiboldsgriin, Germany |  | 2.5% mortality, 70-73% ameliorations | **71.5%** |
| Warren (2006)(8) | Turban (Davos), Switzerland |  | 4.36% mortality, 40% cures, 40% ameliorations | **80.0%** |
| Warren (2006)(8) | Hohenbornef, Switzerland |  | 14.51-28.91% cures | **21.7%** |
| Warren (2006)(8) | Nordrach, Germany |  | 30% cures, 65% ameliorations | **95.0%** |
| Warren (2006)(8) | Ehalila, Finland | This sanatorium treated poor patients | 13.5% mortality, 36.7% cures, 33% ameliorations, 16.7% not ameliorated | **69.7%** |
| Warren (2006)(8) | Falkenstein, Germany | These outcomes are for poor patients specifically, and only early cases were admitted to this sanatorium | 13% cures, 77% not ameliorated | **13.0%** |
| Warren (2006)(8) | Canigou, France |  | 43.8% cures | **43.8%** |
| Warren (2006)(8) | Adrirondack Cottage, United States of America | Patients pay two-thirds of expenses | 20-25% cures, 30-35% ameliorations | **55.0%** |
| Warren (2006)(8) | Winyale, United States of America | Recommends that patients stay 6 months to two years | 4.1% mortality, 22.64% cures, 42.47% ameliorations | **65.1%** |
| **Average percentage of patients that improved:** | | | | **65.5%** |

^a^ Original notation in Warren 2006(8).

**References:**

1.	Ascoli V. The anti-tuberculosis movement in Italy. British Journal of Tuberculosis. 1912;6(2):71–9.

2.	Curran J. The sanatorium patient. Irish Journal of Medical Science (1926-1967). 1942;17(4):113–27.

3.	Eylers E. Planning the Nation: the sanatorium movement in Germany. The Journal of Architecture. 2014 Sep 3;19(5):667–92.

4.	Lawson D. Consumptive Sanatoria: Are They Worth While? Glasgow Med J. 1908;69(3):161–78.

5.	McCrindle JD. Some recent experiences of the sanatorium in dealing with tuberculosis. Public Health. 1912;26:41–8.

6.	Porter GD. Anti-Tuberculosis Work in Canada. Trans Am Climatol Clin Assoc. 1921;37:185–92.

7.	Rucker WC, Kearny RA. Tuberculosis in Switzerland: Results of the Campaign against the Disease. Public Health Reports (1896-1970). 1913 Nov 27;28(52):2815–29.

8.	Warren P. The evolution of the sanatorium: the first half-century, 1854-1904. Can Bull Med Hist. 2006;23(2):457–76.
